# Survivin shRNA重组慢病毒构建及对A549细胞增殖的影响

**DOI:** 10.3779/j.issn.1009-3419.2011.12.01

**Published:** 2011-12-20

**Authors:** 丽红 武, 金河 王, 茜 王, 懂懂 贾, 建琴 梁

**Affiliations:** 100091 北京，中国人民解放军第309医院结核病研究所 Tuberculosis Research Institute, the 309^th^ Hospital of Chinese PLA, Beijing 100091, China

**Keywords:** 慢病毒, Survivin, 短发夹RNA, 增殖, Lentivirus, Survivin, shRNA, Proliferation

## Abstract

**背景与目的:**

Survivin是凋亡抑制蛋白(inhibitor of apoptosis protein, IAP)家族的成员, 在多种肿瘤组织中高度表达而在终末分化细胞中极少表达, 因此可以作为癌症治疗的理想靶点。本研究旨在通过构建*Survivin*基因shRNA的慢病毒质粒并干扰肺癌细胞A549中Survivin的表达, 分析其对细胞增殖的影响。

**方法:**

设计Survivin干扰靶序列, 构建重组质粒; 将pLL3.7-Survivin转染293T细胞后利用Hela细胞检测病毒的滴度并感染A549细胞, 应用RT-PCR和Western blot检测干扰效果; MTT与流式细胞术分析其对细胞增殖的影响。

**结果:**

本研究成功构建了重组质粒; 重组质粒可抑制A549细胞中*Survivin*基因的表达; 细胞受阻于G_2_/M期。

**结论:**

本研究构建的重组质粒可抑制*Survivin*基因的表达并影响细胞的增殖, 其为研究RNAi介导的肺癌基因治疗打下基础。

## Abstract (en)

**Background and objective:**

Survivin is a member of the inhibitor of apoptosis family of proteins.The Survivin protein is highly expressed in most human tumors, but it is completely absent in terminally differentiated cells.Consequently, Survivin is an ideal target for cancer therapy because cancer cells are targeted and normal cells are left alone.The aim of this study is to construct a lentivirus-shRNA vector, and to disrupt the expression of Survivin in A549 cells.The effect of sh-RNA Survivin on A549 cells was analyzed.

**Methods:**

Target DNA sequences of Survivin shRNA were designed to obtain recombinant plasmids.After the plasmids were transfected into 293T cells, the virus was collected.Hela cells were used to detect the virus titer.Survivin mRNA and protein expression in the infected A549 cells were detected via reverse transcription polymerase chain reaction and Western blot analysis.The proliferation of A549 cells were detected via 3-(4, 5-dimethylthiazolyl)-2, 5-diphenyltetrazolium bromide and flow cytometry assays.

**Results:**

The recombinants were successfully constructed, and Survivin expression was inhibited.The cells were blocked at the G_2_/M phase.

**Conclusion:**

Recombinant lentivirus with shRNA targeting Survivin was successfully constructed.The lentivirus can down-regulate Survivin expression in A549 cells as well as inhibit proliferation, and is hence a potential gene therapy for lung cancer.

Survivin又名BIRC5, 其是一种凋亡抑制蛋白, 是凋亡抑制蛋白(inhibitor of apoptosis protein, IAP)家族的成员, 其通过抑制caspase活化而抑制凋亡或程序性细胞死亡。Survivin蛋白通常在人类胚胎组织和肿瘤细胞中高表达^[1]^, 同时其表达受细胞周期的调控, 仅在G_2_/M期高表达^[2]^。RNAi是在转录水平上控制基因表达的新技术^[3]^。本研究通过利用shRNA以慢病毒为表达载体沉默*Survivin*基因, 研究其对A549细胞增殖的影响, 从而为肺癌的基因治疗提供思路。

## 1 材料与方法

### 1.1 材料

慢病毒系列质粒—核心质粒pLL3.7以及包装质粒pVSVG、pRSV-REV、pMDLg/pRRE均为本实验室保存; 293T细胞、Hela细胞和人肺癌细胞A549由协和医科大学基础医学细胞中心提供; 限制性内切酶HpaI、XhoI、XbaI、NotI和T4连接酶购自New Englang Biolabs; StarFect High-efficiency Transfection Reagent购自GenStar; 超滤离心管购自Millopore; 总RNA提取试剂盒购自LC Sciences; Titan One Tube RT-PCR kit购自Roche; Survivin antibody、GAPDH、tubulin均购自Santa Cruz; mouse-HRP购自北京中杉金桥生物有限公司; ECL购自GE; 流式细胞仪型号为Cytomics FC 500 Beckman Coulter。

### 1.2 方法

#### 1.2.1 Survivin shRNA重组慢病毒的构建及鉴定

根据homo spacies Survivin mRNA序列利用在线软件设计2个19 bp的靶基因序列, 分别为5′GACCACCGCATCTCTACA3′和5′GGCTGGCTTCATCCACTGC3′; Control靶序列为5′GCGAATTTAGGATAATCTC3′。在设计的寡核苷酸序列中, 5′端为*HpaI*酶切位点, 3’端为*XhoI*酶切位点, 中间为9个碱基的loop结构:TTCAAGAGA。序列由Invitrogen公司合成。寡核苷酸退火后与经*HpaI*、*XhoI*线性化的pLL3.7质粒连接, 转化DH5α经氨苄青霉素(Amp)平板筛选。挑取单克隆菌落质粒小提, *XbaI*、*NotI*酶切鉴定同时送Invitrogen公司测序。

#### 1.2.2 细胞培养及转染

293T细胞、Hela细胞和人肺癌细胞A549培养在DMEM培养基中(含10%胎牛血清和1%双抗—青霉素与链霉素)。转染293T细胞前24 h接种适量细胞至转染时细胞密度60%为宜(6 cm培养皿)。转染前1 h-4 h更换新鲜培养基, 细胞均于37 ℃含5%CO_2_的恒温培养箱内培养。取10 μg DNA(pLL3.7:pVSVG:pRSV-REV:pMDLg/pRRE=3:1:1:1)加入PBS至200 μL, 轻轻混匀, 室温放置; 取8 μL StarFect加入PBS至总体积200 μL, 轻轻混匀, 室温放置5 min; 将稀释的StarFect逐滴加入稀释的DNA溶液中, 轻轻混匀, 室温放置15 min; 将上述混合液逐滴加入培养基中, 轻轻混匀。置于37 ℃含5%CO_2_的恒温培养箱内培养, 48 h后收集病毒(转染后6 h换新鲜培养基)。

#### 1.2.3 Survivin shRNA病毒收集及滴度测定

转染6 cm×6 cm培养皿, 48 h后收集培养基, 0.45 μm滤膜过滤, 收集上清加入到超滤离心管中, 5, 000 rpm离心, 过滤至终体积为200 μL, -80 ℃保存备用。感染病毒12 h-24 h前接种4×10^5^个Hela细胞于6孔板中, 并置于37 ℃含5%CO_2_的恒温培养箱内培养。分别用DMEM培养基将病毒按1×10^-1^、1×10^-2^、1×10^-3^、1×10^-4^进行梯度稀释, 同时加8 μg/mL的聚凝胺。吸去上清, 分别加入不同稀释度的病毒液体, 每个浓度重复1次, 同时设置不加病毒的阴性对照。48 h后观察GFP的荧光表达率, 选取荧光表达率1%-10%的稀释度计算病毒滴度。病毒滴度=接种细胞数×稀释度×荧光细胞百分比。

#### 1.2.4 RT-PCR与Western blot

A549细胞感染48 h后按照总RNA提取试剂盒说明书提取RNA, 并进行RT-PCR。Survivin引物序列:Forward:5′CCCTGCCTGGCAGCCCTTTC3′, Reverse:5′CTGGCTCCCAGCCTTCCA3′, 扩增长度为188 bp。GAPDH引物序列:Forward:5′AGAAGGCTGGGGCTCAGGTG3′; Reverse:5′AGGGGCCATGGACAGTCTTC3′, 扩增长度为258 bp。PCR扩增条件为:95 ℃ 1 min, 94 ℃ 1 min, 57 ℃ 30 s, 72 ℃ 30 s, 30个循环; 72 ℃ 10 min。取PCR产物3 μL进行2%琼脂糖凝胶电泳检测。图像采用Quantity One软件分析灰度值。

A549细胞感染48 h后提取总蛋白, 考马斯亮蓝G250法测定蛋白浓度后进行12%SDS-PAGE。72 V转膜2 h, 一抗Survivin和tubulin 4 ℃过夜, 洗膜后室温二抗1 h。ECL显影。

#### 1.2.5 MTT与流式细胞术

将A549细胞以每孔1, 000-10, 000个细胞接种到96孔板, 每孔体积200 μL。细胞分为PBS组、实验组与阴性对照组; 病毒悬液按照1×10^-2^稀释度加入。每组设3个平行。第2天时加病毒, 置培养箱中培养24 h、48 h、72 h。每孔加MTT溶液(5 mg/mL, 用PBS配制, pH7.4)20 μL。继续孵育4 h。弃去培养基, 每孔加150 μL DMSO, 脱色摇床振荡10 min, 使结晶物充分溶解。选择490 nm(570 nm)波长, 在酶联免疫监测仪上测定各孔光吸收值, 记录结果, 以时间为横坐标, 吸光值为纵坐标绘制细胞生长曲线。实验重复3次。

收集感染病毒48 h的A549细胞于离心管中, 1, 000 rpm离心3 min, 弃上清液, 轻弹细胞沉淀物。用5 mL PBS重悬细胞, 离心洗1遍, 弃上清液, 重复同样操作1次。轻弹细胞沉淀物, 约0.2 mL-0.3 mL。固定:用细滴管将以上细胞悬液迅速注入4 ℃预冷的70%酒精中, 轻轻混匀, 4 ℃冰箱保存至少24 h。洗去固定剂:1, 000 rpm离心3 min, 去上清, 轻轻弹起细胞沉淀物, 加PBS后将细胞悬液轻轻混匀。1, 000 rpm离心3 min。RNA酶消化:去上清, 取0.5 mL细胞悬液, 加入RNAse A, 终浓度为50 μg/mL, 37 ℃孵育30 min。将样品管插入冰浴中, 停止RNAse A的消化作用。碘化丙啶(PI)染色:冷却后每个样品加入PI至终浓度为50 μg/mL, 在室温中避光染色至少2 h。用300目(孔径约40 flm-50 flm)尼龙网过滤后, 即可上机测量。

#### 1.2.6 统计学分析

采用SPSS 11.5统计软件进行分析。3次独立实验的数据采用Mean±SD表示, 实验组与对照组的组间差异比较采用*t*检验分析, *P* < 0.05为有统计学差异。

## 2 结果

### 2.1 重组慢病毒质粒鉴定

将质粒用*XbaI*、*NotI*酶切。因插入片段的影响, 重组质粒与未重组质粒约有50 bp的差异(图 1)。测序结果显示质粒构建成功。

**1:**
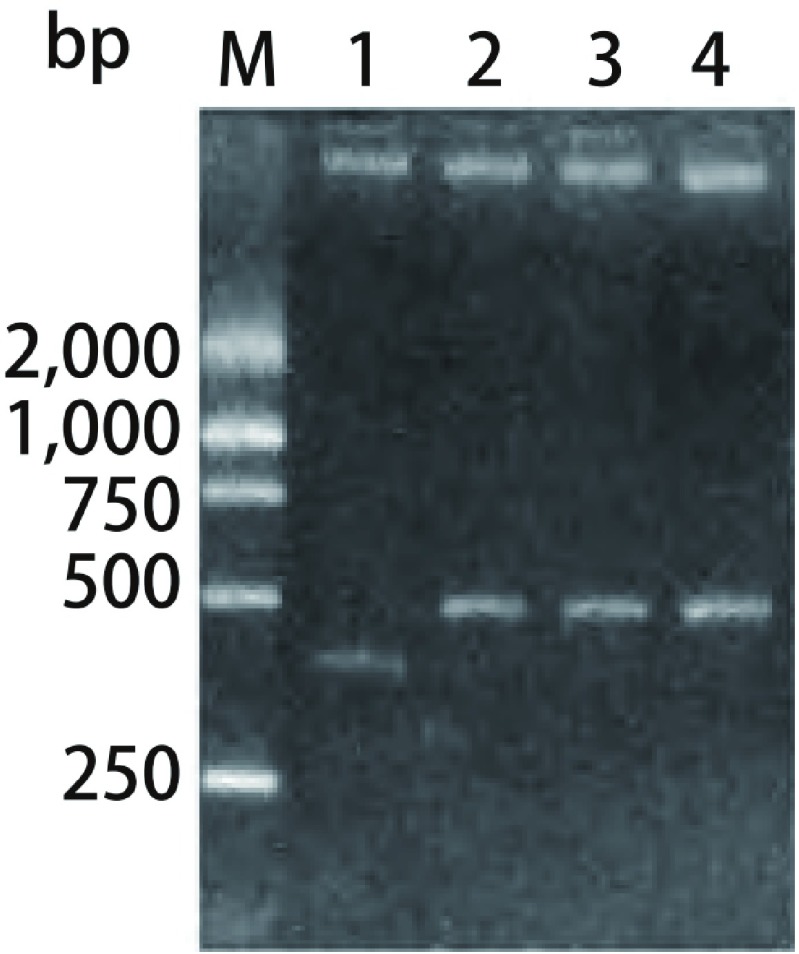
重组质粒双酶切结果 Double enzyme cutting result of recombinant plasmids.M:Marker; 1:pll3.7;2:pll3.7-Control; 3:pll3.7-Survivin1;4:pll3.7-Survivin2.

### 2.2 滴度测定

质粒转染293T细胞, 48 h后荧光显微镜下观察到转染效率 > 90%(图 2A)。Hela细胞感染慢病毒后在1×10^-1^、1×10^-2^、1×10^-3^中观察到有绿色荧光, 慢病毒的滴度约为10^8^ pfu/mL。图 2B为1×10^-2^稀释度感染Hela细胞的效果。

**2:**
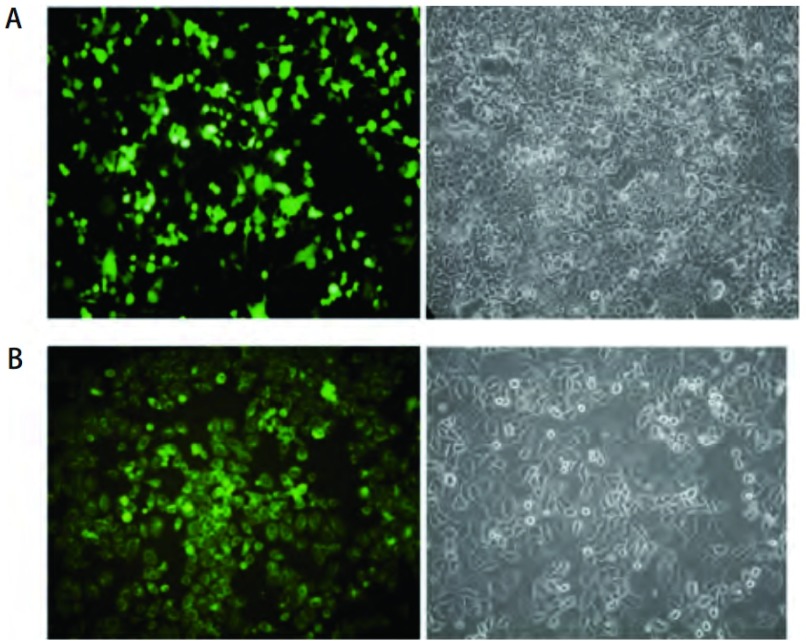
转染效率及病毒滴度检测结果。A:荧光检测转染的293T细胞(左)和明场观察(右); B:荧光检测感染Hela细胞(左)和明场观察(右)。 Results of transfection efficiency and virus titer.A:293T cell shot by fluorescent light (left) and light (right); B:Hela cell shot by fluorescent light (left) and light (right).

### 2.3 干扰效果测定

RT-PCR结果显示shRNA组中*pLL3.7-Survivin2*基因含量明显降低(图 3A), 说明在慢病毒的介导下, shRNA使A549细胞中Survivin mRNA的表达量明显减少。同样Western blot的结果也证实了Survivin蛋白减少的现象(图 3B)。

**3:**
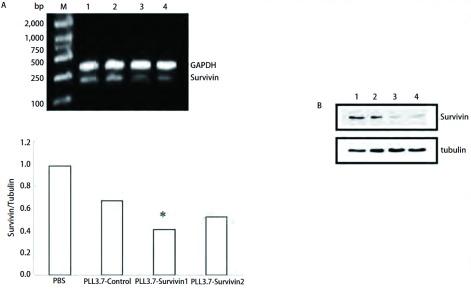
RT-PCR及Western blot检测结果。A:RT-PCR检测Survivin mRNA的表达, 柱状图为相对内参GAPDH的灰度值; 与Control组相比, ^*^P < 0.05。B:Western blot检测Survivin表达量。与Control相比, ^*^P < 0.05。 Results of RT-PCR (A) and Western blot (B).A:The result of Survivin mRNA RT-PCR, anlysis of expression compare with GAPDH; ^*^P < 0.05, *vs* Control.B:The result of Survivin expression by Western blot.1:PBS; 2:pLL3.7-Control; 3:pLL3.7-Survivin1;4:pLL3.7-Survivin2.

### 2.4 Survivin shRNA对A549细胞增殖的影响

A549细胞感染慢病毒不同时间之后, MTT法检测细胞增殖。表 1显示Survivin shRNA对细胞的增殖均起到了一定程度的抑制, 且抑制率具有时间依赖性。流式细胞术结果显示感染pLL3.7-Survivin后处于G_2_-M期的细胞明显增多, 但并没有明显的凋亡峰出现(图 4)。

**1:** pLL3.7-Survivin对细胞增殖的抑制作用 Inhibitory effect of pLL3.7-Survivin on cell proliferation

Group	Inhibitory rate (%)		
	24 h	48 h	72 h
PBS	0.35±0.55	0.37±0.97	0.40±0.68
pLL3.7-Control	0.36±0.73	0.41±0.56	0.45±0.32
pLL3.7-Survivin2	31.12±1.98^*^	39.71±2.13^**^	47.56±1.86^**^
^*^P < 0.05;^**^P < 0.01, compared to group PBS.			

**4:**
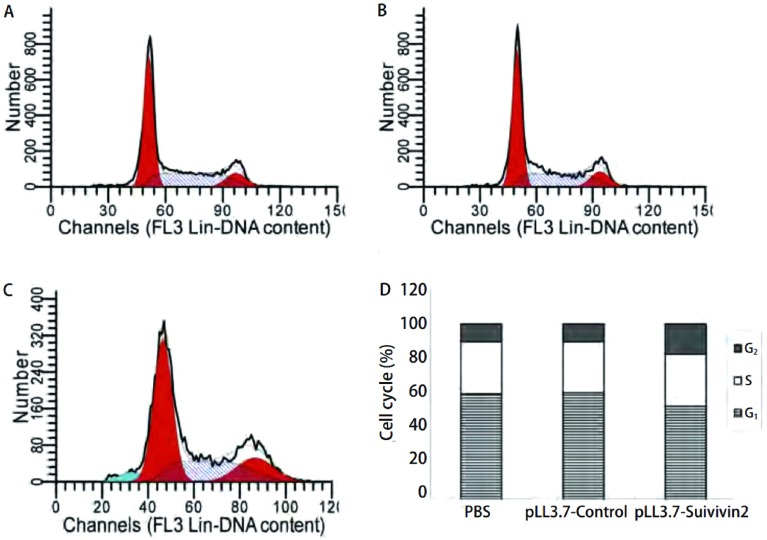
流式细胞术检测细胞周期。 Results of flow cytometry.A:PBS; B:pLL3.7-Control; C:pLL3.7-Suivivin2;D:results of cell cycle.

## 3 讨论

Survivin大小为16.5 kDa, 在体内以二聚体形式存在, 是凋亡抑制蛋白家族成员。其主要在细胞周期的G_2_/M期表达。Survivin与活化的caspase3和caspase7结合, 使caspase聚合体分离从而抑制其活性, 进而保护了细胞周期调节因子如p21, 从而抑制细胞凋亡^[4]^。Survivin可使p21与Cdk4解离, 导致Cdk4活化, 细胞进入增殖周期进而大量细胞无限生长, 从而有利于肿瘤的发生^[5]^。Suvivin在细胞分裂中起重要作用, 它的表达与细胞的周期变化相协调^[6, 7]^。Survivin在G_1_期开始表达增加, G_2_/M期达到峰值。有丝分裂期间Survivin作为染色体乘客复合体的一部分发挥动粒微管的调节作用。Survivin在有丝分裂中期和后期作用于中心体和纺锤体维持稳定性, 确保姐妹染色体单体的准确分离。如果从系统中去除Survivin, 动粒微管系统不能正确形成, 从而细胞停止分裂, 最终导致细胞死亡。在细胞分裂期间Survivin也结合到有丝分裂器的微管, 通过与周期依赖蛋白激酶1(cyclin-dependent protein kinase 1, CDK1)作用, Survivin的Thr34发生磷酸化, 从而稳定蛋白并抑制分裂细胞的凋亡^[7, 8]^。Survivin在正常成年分化组织中不表达或低表达, 而在人类恶性肿瘤组织中表达极高^[9-11]^, 故针对Survivin的基因治疗具有很好的靶向性、特异性和安全性^[12]^。

近几年来, 慢病毒载体因其独特的优势, 逐渐成为表达载体中的热点。慢病毒通常是由HIV去除*env*、*vif*、*vpr*、*vpu*、*nef*等毒性基因改造而来。复制缺陷型载体由VSVG代替HIV的包膜进行包装, 具有单复制周期、安全、宿主范围广的优点^[13]^。

本实验通过设计Survivin干扰靶序列, 构建重组质粒, 并将pLL3.7-Survivin转染293T细胞后利用Hela细胞检测病毒的滴度并感染A549细胞, RT-PCR和Western blot检测干扰效果明显; MTT与流式细胞术分析显示细胞受阻于G_2_/M期。这为研究RNAi介导的肺癌基因治疗打下了基础。
